# Pomological, Sensorial, Nutritional and Nutraceutical Profile of Seven Cultivars of Cherimoya (*Annona cherimola* Mill)

**DOI:** 10.3390/foods10010035

**Published:** 2020-12-24

**Authors:** Carla Gentile, Giuseppe Mannino, Eristanna Palazzolo, Giuseppe Gianguzzi, Anna Perrone, Graziella Serio, Vittorio Farina

**Affiliations:** 1 Department of Biological, Chemical and Pharmaceutical Sciences and Technologies (STEBICEF), University of Palermo, Viale delle Scienze, 90128 Palermo, Italy; anna.perrone@unipa.it (A.P.); graziella.serio01@unipa.it (G.S.); 2 Department of Life Sciences and Systems Biology, Innovation Centre, Plant Physiology Unit, University of Turin, Via Quarello 15/A, 10135 Turin, Italy; giuseppe.mannino@unito.it; 3 Department of Agricultural, Food and Forest Sciences (SAAF), University of Palermo, Viale delle Scienze, 90128 Palermo, Italy; eristanna.palazzolo@unipa.it (E.P.); giuseppe.gianguzzi@unipa.it (G.G.); vittorio.farina@unipa.it (V.F.)

**Keywords:** polyphenols, sensory analysis, mineral content, proanthocyanidins, carotenoids, antioxidant activity, FRAP, DPPH, ABTS, CAA

## Abstract

In this work, the food quality of four international (*Campas*, *Chaffey*, *Fino de Jete* and *White*) and three local (*Daniela*, *Torre1* and *Torre2*) cultivars of Cherimoya (*Annona cherimola* Mill) was investigated. With this aim, pomological traits, sensorial attributes, physiochemical parameters (pH, total soluble content and total acidity), nutritional composition (macro- and micro-nutrients) and nutraceutical values (bioactive compounds, radical scavenging and antioxidant properties) were evaluated. Among the seven observed cultivars, *Fino de Jete* was identified as the best, not only for its commercial attributes such as pomological traits and physiochemical values, but also for its nutritional composition. On the other hand, *Chaffey* and *Daniela* were the cultivars with the highest content of polyphenols, proanthocyanidins, and with the strongest antioxidant capacity. Concerning the two local ecotypes, *Torre1* and *Torre2*, they displayed a balanced nutritional profile that, if combined with their discrete nutraceutical, physicochemical and pomological values, may result in a reassessment of their commercial impact. In conclusion, our data provide interesting information about the pomological, nutritional, and nutraceutical properties of cherimoya fruits. Our results, in addition to promoting the commercial impact of local cultivars, may increase the use of individual cultivars in breeding programs.

## 1. Introduction

The *Annonaceae* Juss. family covers more than 2000 species, of which 120 belong to the genus *Annona* L. [1]. The most famous species are *Annona cherimola* Mill (cherimoya), *Annona muricata* L. (soursop), *Annona squamosa* L. (conde fruit), *Annona reticulata* L. (custard apple), and the interspecific hybrid *Atemoya* (*A. cherimola* × *A. squamosa*). In particular, *Annona cherimola* is the most diffused specie in subtropical countries. It is an indigenous tree of Andean South America and it has naturalized in tropical highlands and subtropical areas of South America [2]. The marketable value of cherimoya is related to its big, heart-shaped and conical fruit [3], which may reach considerable weight and size. The edible flesh of these fruits is white, creamy, and with a custard-like consistency [4]. The aromatic flavor is a mix of papaya, banana, pineapple, and passion fruit [5]. Although cherimoya fruit is consumed as fresh fruit, it can be also processed making yogurt, ice creams and other desserts. It is not recommended to ripen the fruits on the tree, because they lose quality. Cherimoya fruits are generally harvested when not fully mature, and left for ripening under controlled storage conditions. The time of harvesting is commonly determined by the changes of skin fruit color, which turn from green to yellowish-green in the proximity of physiological maturity [6]. In Europe, Spain is the most important producer of cherimoya fruits, and the most important cultivars are *Campas* and *Fino de Jete*, which are also the most widespread cultivars in the global market. In Italy, *A. cherimola* is well adapted to the pedoclimatic conditions of the tyrrhenian coastal areas of Sicily and Calabria, where there are good climate conditions for the production of other exotic and tropical fruits, such as loquat, mango, litchi, avocado, banana and papaya [7,8,9,10]. In particular, in Sicily, in addition to affirmed cultivars such as *Fino de Jete*, local ecotypes are also cultivated with very limited diffusion. Concerning the nutritional value, cherimoya flesh possesses a high content of sugars, while having low fat content and, in comparison with other tropical fruits, also good Ca and P content [11]. However, although the nutraceutical properties of other *Annona* species have been extensively studied, those related to cherimoya fruits are much less investigated. The majority of the available literature data suggests that this tropical fruit is an interesting source of bioactive compounds, including polyphenols (catechin, proanthocyanidins, hydroxytyrosol) [12,13], alkaloids (annocherines, norisocorydine, cheritamine, annonaine) [14,15], acetogenins (cherimolin-2 and almunequin) [16], terpenes (myrcene, pinene, linalool, caryophillene, terpenolene and germacrene) [17] and cyclopeptides (cherimola cyclopetide E and cherimola cyclopetide F) [18,19]. In addition, antioxidant [20], pro-apoptotic [3,16,17,21], anti-protozoal [22], and anti-diabetic [23] activities were also demonstrated for extracts obtained from different part of the fruit. 

The aim of this study was the investigation of the pomological, physiochemical, sensorial, nutritional and nutraceutical attributes of seven cultivars of cherimoya fruits grown in Sicily. Our results provide comprehensive information on the quality of cherimoya fruits and can be useful for the improvement of the utilization of the specific genotype.

## 2. Materials and Methods 

### 2.1. Plant Material

The fruits were obtained from trees grown in Vivai Torre (Milazzo, Sicily, Italy; 38°19**′** N, 15°24**′** E; 20 m a.s.l.). Four international affirmed CVs (*Campas*, *Chaffey*, *Fino de Jete* and *White*) and three local CVs (*Daniela*, *Torre1* and *Torre2*) were selected (Table 1). The fruits of each CV were picked from three 15-year-old trees, grafted on their own rootstock. The trees were planted in North-South direction, with an inter-tree spacing of 6 m and 6 m between rows. The yield per tree was measured by weighing and counting the total number of fruits per tree, and at each harvest time, the trunk circumference was measured at ~15 cm above the graft union. The yield efficiency and crop load were expressed in kilograms or number of fruits per trunk of a cross-sectional area (TCSA) or leaf area. A sample of 30 fruits per CV (10 fruits per 3 trees) was hand-picked when not fully mature, and the color changed from green to yellow. After harvest, the fruits were left to ripen under storage conditions (20 °C). Fifteen fruits were employed for pomological and physicochemical analysis, and 5 of them were employed for the sensorial evaluation. Finally, the others 15 fruits were immediately frozen in liquid nitrogen and then stored at −80 °C until the analysis of nutrients, bioactive compounds, and antioxidant activity was performed within 3 months. Immediately before the analysis, three fruits for each CV were thawed and peeled, and then the seeds were removed. The chopped pulp was homogenated. Three aliquots of each homogenate for each analysis were employed.

### 2.2. Pomological and Physiochemical Analysis

Fruit weight (FW), longitudinal diameter (LD), transversal diameter (TD), seed weight (SW), peel weight (PeW), pulp weight (PW), flesh firmness (FF), total soluble solids content (TSSC), and titratable acidity (TA) were measured. FW, SW, PeW and PW (g) were determined using a digital scale (Gibertini EU-C 2002 RS, Novate Milanese, Italy); LD and TD (mm) using a digital calliper TR53307 (Turoni, Forlì, Italy); FF (kg/cm^2^) using a digital penetrometer TR5325 with a 8 mm diameter tip (Turoni, Forlì, Italy); TSSC (°Brix) using a digital refractometer Atago Palette PR-32 (Atago Co., Ltd., Tokyo, Japan) and TA (g citric acid per L) using a CrisonS compact tritator (Crison Instruments, SA, Barcelona, Spain). Skin and flesh colors were calculated using a Konica Minolta Colorimeter based on the CIELAB system that measured the lightness (L*) and the variation from red (+a*) to green (−a*), and from green (+b*) to yellow (−b*) in the fruits.

### 2.3. Sensory Analyses

A trained panel consisting of 10 judges (5 females and 5 males, between 22 and 45 years of age) performed the sensory profile analysis, as previously reported [7]. All panelists were trained and developed wide expertise in sensory evaluation of tropical fruits. The judges in preliminary sessions generated 24 sensory descriptors (Table 2), and they evaluated samples using a hedonic scale, assigning to each descriptor a score from 1 (absence of the sensation) to 9 (highest intensity). The order of each sample was randomized for each panelist, and water was provided for rinsing between the different samples.

### 2.4. Nutritional Parameters

#### 2.4.1. Content of Carbohydrates, Lipids, Proteins, Water and Ashes

The carbohydrate and protein content were evaluated as previously described, respectively using Anthrone’s [24] and Kjedahl’s [25] methods. Ash and water contents were determined through the procedure described in AOAC [26]. The content of lipids was calculated after lipid extraction with a gravimetric method, as previously described [27]. Data were expressed as g per 100 g of Pulp Weight (PW).

#### 2.4.2. Mineral Content

The contents of K, Na, Ca, Mg, Fe, Cu, Mn, and Zn were determined by atomic absorption spectroscopy following wet mineralization, and using the instrumental condition as previously described [28]. Briefly, the samples were digested, and approximately 100 mg of dried sample was weighed and incubated with 9 mL of 65% (*w/w*) HNO_3_, and 1 mL of 30% (*w/v*) H_2_O_2_ were added. The temperature was set at 200 °C for 20 min. Once cooled, the digested samples were diluted to a final volume of 50 mL with distilled H_2_O. All measurements were performed using an Agilent 4200 MP-AES fitted with a double-pass cyclonic spray chamber and OneNeb nebulizer. The calibration standards were prepared by diluting a 1000 mg/L multi-element standard solution (Sigma Aldrich and Scharlab S.L.) in 1% (*v/v*) HNO_3_. Finally, P was determined using a colorimetric method [29]. Data were expressed as mg per 100 g of Fresh Weight (PW).

#### 2.4.3. Vitamin Content

Retinol (Vit. A), Riboflavin (Vit. B2), Niacin (Vit. B3), and Ascorbic Acid (Vit. C) were extracted and determined according to previously reported methods. Briefly, Vit. A was extracted and quantified using a commercial kit (Vitamin A Food ELISA Kit, Crystal Chem, NL) and following the manufacturer’s instructions. Vit. B1 and Vit. B2 were respectively extracted using 0.1 N HCl [30] or a solution of 1% (*v/v*) H_2_SO_4_ [31]. Quantification was performed via HPLC equipped with a fluorimetric detector [30,31]. Finally, Vit C was extracted with 10 mL of 1% (*v/v*) HPO_3_ for 45 min from dried extract, previously prepared [7]. After filtration, 1 mL was mixed with 9 mL of C_12_H_7_NC_l2_O_2_ and the absorbance was measured at 515 nm against a blank after 30 min. Vitamin C was quantified using a calibration curve of authentic L-ascorbic acid (0.02–0.12 mg/100 g). Data were expressed as mg per 100 g of Fresh Weight (PW).

### 2.5. Bioactive Compounds and Antioxidant Activities

#### 2.5.1. Total Carotenoid Content (TCC)

TCC was determined in flesh homogenates via spectrophotometric analysis after extraction of carotenoids, as previously reported [8]. Data were expressed as β-carotene Equivalent per 100g of PW, using the molecular weight (536.87 g mol^−1^) and the molar extinction coefficient (2505 M^−1^ cm^−1^) of β-carotene in Hexane.

#### 2.5.2. Preparation of Fruit Extracts

Three aliquots of each homogenate were extracted twice with 70% (*v/v*) EtOH using a 1:20 (*w/v*) ratio. After centrifugation (10 min at 10.000 *g*, 4 °C) and filtration through a Millex HV 0.45 μm filter (Millipore, Billerica, MA), the supernatants were recovered and combined together. Ethanolic extracts were used both for the determination of bioactive compounds and the antioxidant properties.

#### 2.5.3. Total Polyphenol Content (TPC)

The phenolic content of the flesh of the observed CVs of *A. cherimola* was determined in ethanolic extracts via the Folin-Ciocalteu method, with some minor changes as previously reported [32,33]. Results were expressed as mg Gallic Acid Equivalents (GAE) per 100 g of PW.

#### 2.5.4. Determination of the Total ProAnthocyanidins Content (TPAC) and Investigation of the Polymerization Linkage via HPLC-MS/MS

The proanthocyanidins (PACs) were evaluated in the ethanolic extracts via BL-DMAC assay [34] with some minor changes, as previously reported [35]. The PAC concentration in the extracts was expressed as mg PAC-A equivalent per 100 g of PW. 

In order to investigate PAC grade and type, polymerization-binding of catechins was investigated via High-Pressure Liquid Chromatography (HPLC, Agilent 1260, Technologies, Santa Clara, CA, USA) coupled with 6330 Series Ion Trap (Agilent Technologies, USA), as previously reported [9].

#### 2.5.5. Radical Scavenging and Metal Ion Reducing Activity

The antioxidant activity of the ethanolic extracts was measured evaluating both the radical scavenging activity via ABTS [36] and DPPH [37], and the reducing antioxidant power via FRAP [38] assays. Data were expressed as mmol Trolox Equivalent (TE) per 100 of PW as previously reported [35].

#### 2.5.6. Cellular Antioxidant Activity Assay (CAA)

The CAA assay was performed as previously described by Wolfe at al [39], with some minor changes [40]. For the experiments, we used HepG2 (human liver cancer cell line), obtained from American Type Culture Collection (ATCC) (Rockville, MD, USA). The antioxidant activity was expressed as CAA_50_ that is the amount of flesh in cell medium necessary to obtain the 50% of inhibition of oxidative stress, with respect to the positive control. CAA_50_ was calculated from concentration-response curves using linear regression analysis, and it was expressed as µg of PW per mL of cell medium.

### 2.6. Statistical Analysis

Each assay was repeated three times. All data were tested for differences between the CVs using one-way analysis of variance (ANOVA; general linear model) followed by Tukey’s multiple range test for *p* ≤ 0.05, marking significant differences among the samples with different lowercase letters. Principal Component Analysis (PCA) and HeatMap Cluster Analysis were performed using covariant matrix of extraction and varimax rotation. All statistical analyses were performed using SPSS ver. 24. The nucleotide sequences were analysed via CLC software, and the cladogram of gene sequences was performed with ClustalX software by using the Neighbour Joining (NJ) method. Bootstrap values were calculated from 100 resampling of the alignment data. 

## 3. Results and Discussion

### 3.1. Pomological and Physiochemical Parameters

The fruits of the observed *A. cherimola* CVs showed wide variability of the pomological (Table 3) and physiochemical (Table 4) parameters. All the observed CVs, except *Campas* and *White*, reached a considerable size, with a small incidence of SW and PeW on the total FW. For these CVs, the edible part ranged between 73% (*Daniela*) and 87% (*Fino de Jete*) of the total FW. Moreover, *Fino de Jete* and *Daniela* produced the largest and biggest fruits. On the other hand, *Campas* and *White* produced the smallest fruits, with an incidence of non-edible part of about 40%. In addition, significant differences in FF among the different CVs were not recorded. Concerning the edible part, generally, the largest fruits showed also the highest percentage of flesh (PW/FW). The highest yield per tree was obtained in *Daniela* and *Fino de* Jete, whereas *White*, *Campas* and *Torre 2* showed very low values (Table 1). Yield improvement was caused by the increase of the fruit size, rather than to the number of fruits; nevertheless, crop load was higher for *Fino de Jete* and *Chaffey*. Moreover, the highest yield efficiency was observed in *Chaffey* followed by *Fino de Jete.*

The L*, a* and b* parameters of peel and pulp of Cherimoya fruits were minimally influenced by the genotype (Table 4). Low a* and high b* values were recorded in both peel and pulp for all the observed CVs, indicating a brown peel color and a yellow color of the pulp. It was previously suggested that during the maturation of *A. crassiflora* fruits, the decrease of a* and the increasing of b* may be related to chlorophyll degradation and carotenoid accumulation, typical of ripening processes [41]. 

Also, TSSC varied low among the analyzed CVs, and the mean value recorded was 19.4 ± 1.82 °Brix, and the highest values were recorded in *Chaffey* and *Torre1* (Table 4). Our results were similar to those of Andrès-Augustin and colleagues who recorded comparable ranges for TSSC in fruits from commercial and local CVs of Cherimoya from Mexico [42]. Concerning TA, the observed CVs may be grouped in two different subgroups: the first one included *Fino de Jete*, *Torre1*, *Torre2* and *White*, showing a TA value more than 4.0 g malic acid per L; the second one included *Campas*, *Chaffey*, and *Daniela*, with a TA value less than 4.0 g of malic acid per L (Table 4). When the TSSC/TA ratio is considered, the fruits of the observed CVs may be divided between the sweetest (*Campas*, *Cheffey* and *Daniela*) and the bitterest (*Fino de Jete* and *White*). On the other hand, *Torre1* and *Torre2*, the two local CVs, displayed intermediate behaviors.

### 3.2. Sensorial Analysis

**Figure 1:**
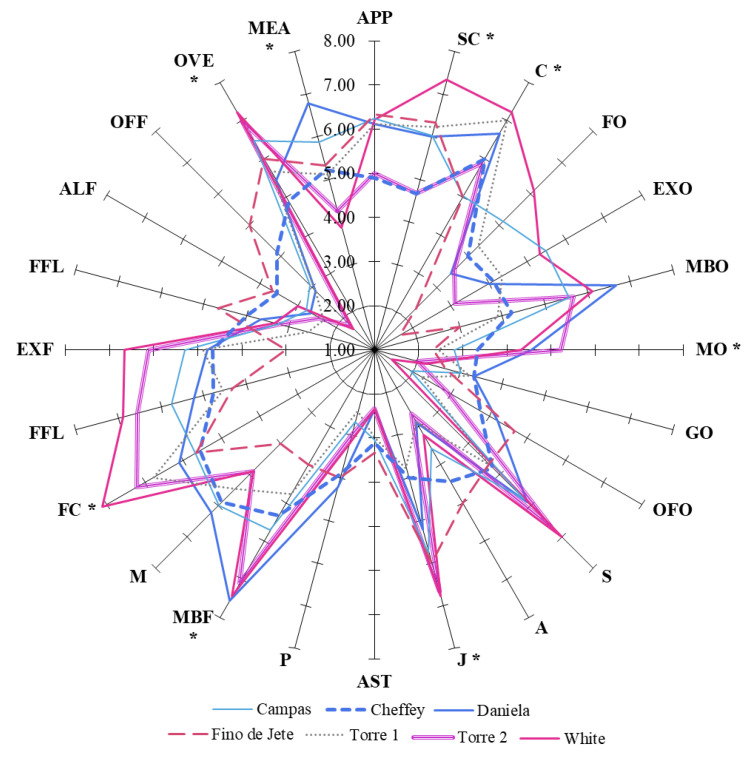
Sensorial descriptors of the observed seven cultivars of *Annona cherimola* fruits. Values are represented as mean ± SD. For each series, the symbol “*” indicate statistical (*p* < 0.05) differences among the different cultivars, as measured by Student’s *t*-test.

The panel evaluation produced sensory profiles indicating that both commercial and local CVs have good organoleptic characteristics for fresh consumption, thanks to the good combination of some key attributes (Figure 1). In particular, the fruits recorded high values for appearance (APP), skin color (SC), flesh color (FC), consistency (C), juiciness (J), and melon, banana, and pear odor (MBO). The combination of these parameters with the low values for the medicine (MO) and grassy odor (GO) resulted in a good fruit appeal for the consumer. 

Concerning the taste, sensorial analysis data showed that the observed fruits had high values of sweetness (S) and low values for astringent (AST), pungent (P), and acid (A) tastes. In particular, acidic taste is a sensorial attribute known to be one of the most important quality traits for the consumer, and its perception may be correlated with the TSSC/TA ratio. However, the lack of significant Pearson correlation between S and TSSC/TA ratio is not surprising since this correlation is typically stronger for more bitter fruits [43]. Sensorial analysis also suggested significant differences among the CVs concerning specific parameters such as juiciness (J), pulp (FC) and peel (PC) color and consistency (C). In particular, panel evaluations suggested that fruits with the lowest FF (*Fino de Jete* and *Torre1*) are those more appreciated because of their consistency.

### 3.3. Nutritional Parameters

The nutritional values per 100 g of the pulp of Cherimoya fruits are shown in Table 5. We recorded a mean moisture content equal to 79.33 ± 1.11 g per 100 g of PW. The mean value for the content of proteins, fats, and sugars was equal to 1.62 ± 0.14, 0.22 ± 0.04, and 14.09 ± 1.23 g per 100 g of PW, respectively. These values are in accordance with those reported from Morton [44]. Nerveless, strong differences in the macronutrients among the observed seven CVs were not measured, *Chaffey* was the CV with the highest sugar content, and *Fino de Jete* the CV with the lowest. 

Additionally, *Fino de Jete* also showed the lowest fat content. Concerning fibers, they were about 3% of the PW, reaching more than 4% in *Campas*, *Daniela*, *Fino de Jete*, and *Torre1*. Our results, in agreement with other literature data, demonstrated that cherimoya is a tropical fruit with high nutritional value. Indeed, it had low fat content, while containing an amount of sugars and proteins generally higher than other common tropical fruits, including mango [7], kiwi [45], pineapple [46], and papaya [10]. 

Micronutrients, including minerals and vitamins, are involved in several biochemical processes, and their balanced intake is important to prevent deficiency diseases. Plant foods are important sources of these nutrients [47]. The mineral composition in 100 g of PW is reported in Table 5. K was the most abundant mineral in all the analyzed samples, ranging between 25% and 42% of the total mineral content. Moreover, our analysis showed how Cherimoya, as well as other tropical fruits, is a very reach source of Mg, Ca, and P. In particular, our results showed that the observed seven CVs of Cherimoya had an amount of these micronutrients from two- to four-fold higher than other common edible fruits, such peaches [48,49] and apple [50]. The amount of Na recorded had a mean value of 19.09 ± 2.02 mg per 100 g of PW; that is higher than that reported for others tropical fruits, such as banana, guava, mango, papaya and pineapple [51]. Concerning micro-minerals, our results suggest that cherimoya fruits are an extraordinary source of Zn, containing two-fold the amount normally present in red currant [51]. Moreover, we recorded high amounts of Mn and Cu, meanwhile the Fe contents were markedly lower. Globally, except for Ca and Zn content, our analysis showed a mineral content comparable to that obtained by Leterme [47]. Finally, concerning the general mineral composition among the different CVs, our results revealed significant differences only in K content, recording the highest content in *Fino de Jete* and lowest one in *Torre2*. 

Concerning the quantified vitamins, our analysis revealed that the analyzed fruits are a good source of ascorbic acid, with a mean value equal to 37.66 ± 8.41 mg per 100 g of PW. On the other hand, we found a great variability among the observed seven CVs. Indeed, *Torre2* was the CV with the highest content of Vitamin C, recording an amount two-fold higher than *Chaffey*.

### 3.4. Nutraceutical Parameters

#### 3.4.1. Total Phenolic Content

**Figure 2:**
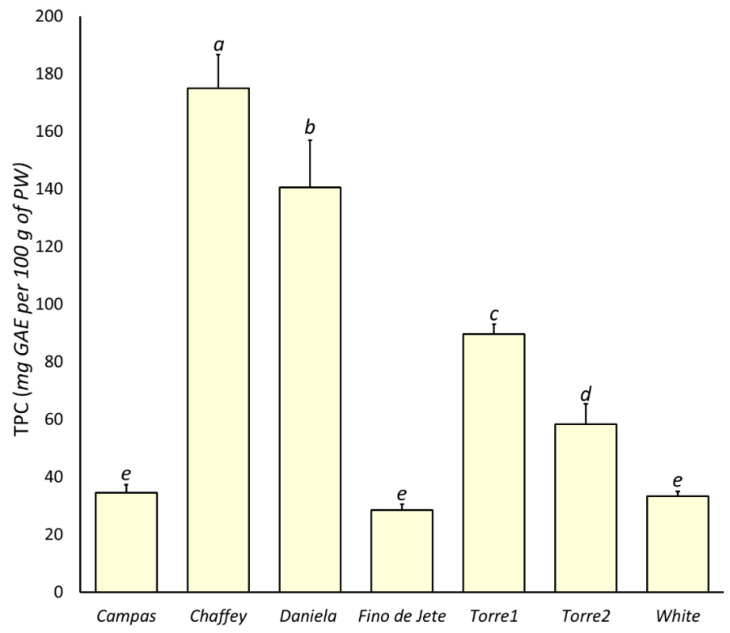
The total polyphenolic content (TPC) in the flesh of the nine observed CVs of *Annona cherimola* fruits measured via Folin-Ciocolteau assay. The bars represent mean ± SD. Different lowercase letters on the top of bars indicate significant differences at *p* ≤ 0.05 as measured by one-way ANOVA followed by Tukey’s multiple range test. The letter “a” denotes the highest value.

Polyphenol compounds are the most abundant dietary phytochemicals [40], and several scientific reports demonstrate their positive influence on human health [9]. On the other hand, several biological actions are documented, including antioxidant [52], antinflammatory [53,54], antidiabetic [55], antiproliferative [3,8], antihypertensive [56], and antihyperlipidemic [57] effects. In this work, TPC in the flesh of the seven observed CVs was measured via Folin-Ciocalteu assay (Figure 2). Our analysis revealed that TPC varied between 28.50 ± 1.92 (*Fino de Jete*) and 174.90 ± 11.69 (*Chaffey*) mg GAE per 100 g of PW, recording an average value equal to 75.18 ± 57.94 mg GAE per 100 g PW. The mean value is higher than those reported for the flesh of other tropical fruits with high commercial impact, including kiwi, papaya, mango and avocado [7,10,45,58]. Furthermore, the mean value for TPC in our fruits was 10-fold higher than for cherimoya fruits from Portugal [59], but 3-fold lower than those obtained for the flesh of fruits from Ecuador [60]. The very large range suggests a significant variability among the analyzed genotypes. Although the contribution of reducing compounds different from polyphenols to TPC value cannot be excluded, the minimal differences in the content of protein and sugar (Table 4 and Table 5) among the seven observed cherimoya fruits suggests that the observed range in TPC mainly depended on a different content of polyphenols. Among the observed CVs, the highest value was observed for *Chaffey*, followed by *Daniela*. On the other hand, the CVs with the highest commercial impact (*Campas*, *White* and *Fino de Jete*) recorded the lowest TPC. Finally, the TPC of the two local ecotypes, *Torre2* and especially *Torre1*, was considerable, and only lower than that recorded for *Chaffey* and *Daniela*.

In our previous work, we also evaluated the phytochemical composition of leaves obtained from the same observed CVs of *A. cherimola* [3]. Comparing TPC values measured in the leaves with that recorded in this work for the flesh of the respective fruits, we found that for all the observed CVs, the leaves contained more polyphenols than the fruits. Moreover, for the leaves, a different ranking for TPC was observed. In particular, *Torre2*, *White*, and *Fino de Jete* were the CVs with the highest TPC in the leaves; meanwhile, *Daniela* and *Torre1* were those with the lowest [3].

#### 3.4.2. Content of Proanthocyanidins

Proanthocyanidins are polyphenols of high molecular weight with documented protective actions for human well-being [61]. In particular, their potential protective effect on the gastrointestinal tract is very interesting. Indeed, thanks both to their high digestive stability at gastrointestinal conditions, and to their reduced intestinal absorption [62,63,64], PACs may reach high concentrations in the intestinal lumen, producing significant biological effects at the local level. The potential benefit of PACs in chronic intestinal inflammation is supported by numerous studies [9,40,65,66,67,68,69,70], and epidemiological data show an inverse correlation between food intake rich in proanthocyanidins and the risk of developing colorectal cancer [9,71,72]. 

**Figure 3:**
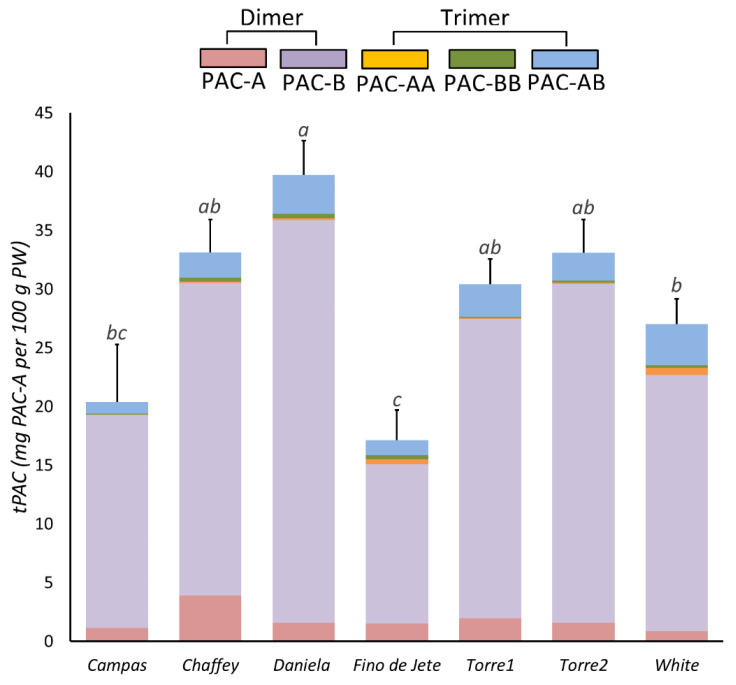
Total proanthocyanidin content (tPACs) in the flesh of the nine observed CVs of *Annona cherimola* fruits measured via BL-DMAC assay. The bars represent mean ± SD. Different lowercase letters on the top of the bars indicate significant differences at the *p* ≤ 0.05 level as measured by one-way ANOVA followed by Tukey’s multiple range test. The letter “a” denotes the highest value. Inside each bar, the different colors indicate the percentage composition of PAC measured by HPLC-MS/MS, as reported in the Materials and Methods section.

We evaluated tPACs in the flesh of the seven observed cherimoya fruits via BL-DMAC assay (Figure 3). We recorded a tPAC content ranging between 10.33 ± 4.51 (*Fino de Jete*) and 51.67 ± 4.04 (*Daniela*) mg PAC-A equivalent per 100 g of PW, with mean values of 28.54 ± 7.98 mg PAC-A equivalent per 100 g of PW. In particular, the highest tPACs was recorded for *Daniela*, followed by *Chaffey*, *Torre2* and *Torre1*; meanwhile, the lowest content was recorded for the *Fino de Jete* and *Campas*.

**Figure 4:**
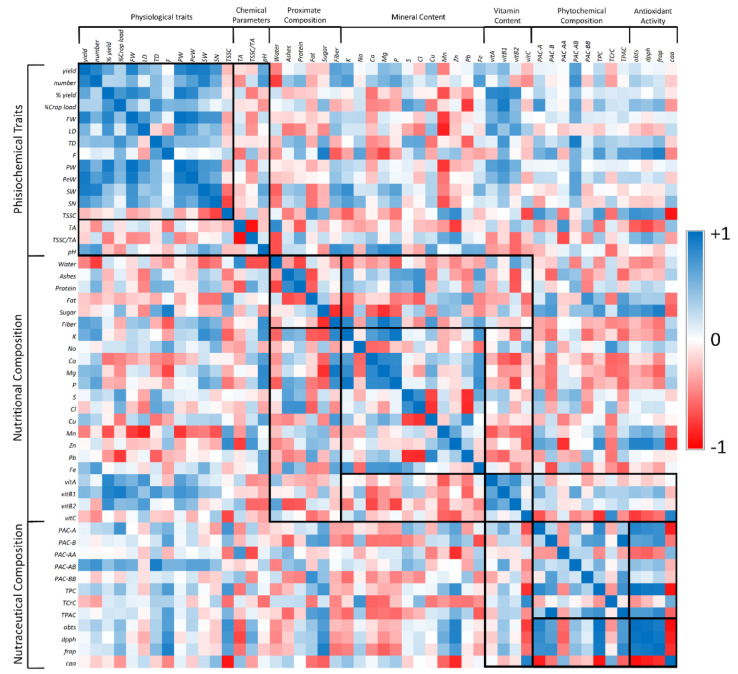
A Pearson’s correlation heat map displaying the correlation coefficient (ρ) based on physiochemical, nutritional, and nutraceutical data of the seven observed cultivars of *Annona cherimola* fruits. Different colors represent the negative (red) to positive (blue) correlation between two different parameters.

Finally, the correlation coefficient (*p* = 0.76), measured by Pearson statistical analysis (Figure 4), suggested that tPCA strongly contributes to the previously measured TPC value. Our analysis is in accordance with García-Salas et al., who evaluated the tPAC and TPC contents in ethanolic extracts from fruits of two different CVs of Cherimoya (*Fino de Jete* and *Campas*) cultivated in Spain [12]. In particular, they showed that cherimoya pulp essentially contains PACs in addition to hydroxytyrosol and traces of luteolin [12].

Concerning the PACs profile, HPLC-MS/MS analysis revealed that in cherimoya fruits, B-type PACs represented about 90% of the total PACs. In particular, *Daniela* displayed the highest percentage, containing more than 94% of B-type PAC; meanwhile, *Chaffey* was the CV with the highest A-type PACs percentage, reaching more than 13%. Even if the greater bioactivity of PAC-A with respect to PAC-B type is well-known [3,9,32,73], the presence of the A-type PAC is very limited in food sources [74,75]. On the other hand, the literature data suggest a higher dosage of PAC-B can exert comparable bioactivity to the PAC-A type [73]. Regarding the polymerization grade of PACs contained in our fruit extracts, we found dimers and a small number of trimers. Our findings are in accordance with Garcia and colleagues, that reported almost exclusively low molecular weight PACs in cherimoya flesh [12]. Although the limited PAC bioavailability, experimental scientific data indicate that their intestinal absorption is inversely related to the polymerization degree [62]. Consequently, dimers and trimers of PAC may also poorly absorbed at the intestinal level [62,76]. Our results would therefore indicate that the cherimoya PAC fraction may be at least partially bioavailable. 

#### 3.4.3. Content of Carotenoids

**Figure 5:**
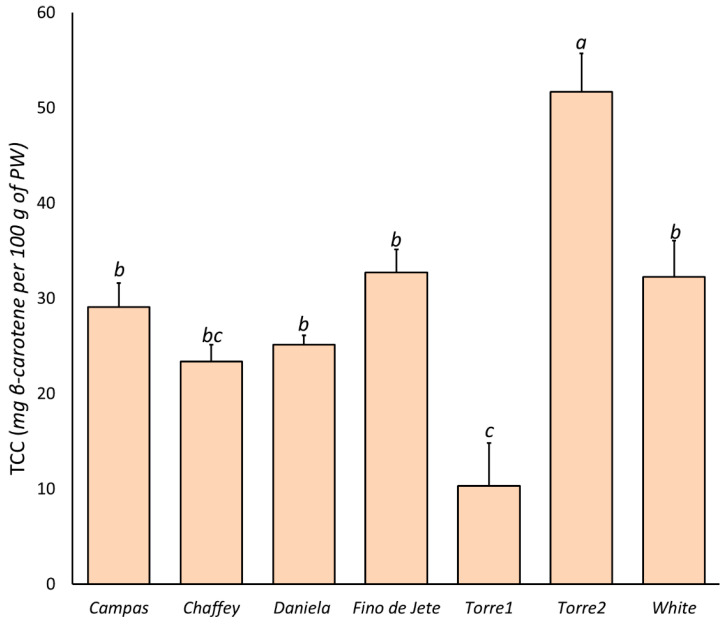
The Total Carotenoid Content (TCC) in the flesh of the nine observed CVs of *Annona cherimola* fruits. The bars represent mean±SD. Different lowercase letters on the top of bars indicate significant differences at *p* ≤ 0.05 as measured by one-way ANOVA followed by Tukey’s multiple range test. The letter “a” denotes the highest value.

Several scientific studies probe how carotenoid intake contributes to preventing human diseases related to oxidative stress [77]. On the one hand, animals are not able to synthesize carotenoids, and plant foods constitute the major carotenoid sources in the human diet [7,77]. The TCC of the seven observed CVs of *A. cherimola* fruits is reported in Figure 5. Our results showed that cherimoya fruits contain very low amounts of carotenoids. Indeed, the TCC ranged between 10.33 ± 4.51 (*Torre1*) and 51.67 ± 4.04 (*Torre2*) μg β-carotene per 100 g of PW, with an average value of 29.22 ± 12.47 μg β-carotene per 100 g of PW. This value is about 100-fold less than that recorded for mango and papaya fruits [7,10]. On the other hand, our results were in accordance with those listed in the USDA National Nutrient Database [78].

#### 3.4.4. Antioxidant Properties

Phytocomponents display various biological properties, and the determination of the potential bioactivity of plant extracts may contribute to their valorization for food fortification, but also be of use in the cosmetic and pharmaceutical fields [3,9]. Frequently, the bioactivity of phytochemicals is related to their antioxidative properties, not only preventing oxidative stress but also being useful for the modulation of important redox-dependent cellular functions [48,79].

**Figure 6:**
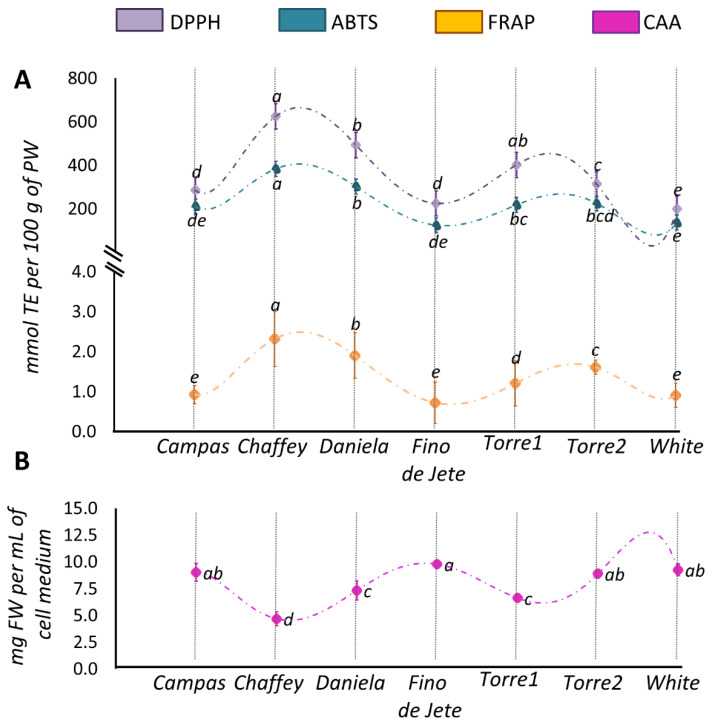
The antioxidant activities of ethanolic extracts of the flesh of the seven observed CVs of *Annona cherimola*. Panel (**A**) shows values measured by the radical scavenging (DPPH = violet; ABTS = light blue) and metal-reducing (FRAP = yellow) assays. Panel (**B**) shows THE CAA_50_ value. Within the same series, different lowercase letters indicate significant difference at the *p* ≤ 0.05 level as measured by one-way ANOVA followed by Tukey’s multiple range test. The letter “a” denotes the value.

In this work, the antioxidant properties of the ethanolic extracts of the flesh of the observed cherimoya fruits were evaluated in in solution assays and in a cell-based lipid peroxidation model. Regarding in solution assays, ABTS and DPPH were used to measure the radical scavenging activity; meanwhile, FRAP was used to evaluate the metal-reducing activity (Figure 6, Panel A). The mean values for the radical scavenging activity evaluated by DPPH and ABTS assay were 363.11 ± 153.59 and 228.75 ± 90.52 mmol TE per 100 g PW, respectively. A lower average value (1.36 ± 0.59 mmol TE per 100 g PW) was measured for the metal-reducing activity via FRAP assay. Peculiar characteristics of the reaction mixtures of the different assays and specific differences in the electronic transfer mechanism may explain the different antioxidant activities recorded [80]. Despite the variability evaluated in the antioxidant activity, the trend among the analyzed CVs was not influenced by the different assays, as suggested by the positive correlation (Figure 4) between the values obtained from the three assays (ρ_ABTS/DPPH_ = 0.968; ρ_ABTS/FRAP_ = 0.917; ρ_DPPH/FRAP_ = 0.949). On the other hand, the obtained results highlight a significant variability in the nutraceutical potential of the analyzed CVs. *Chaffey* and *Daniela* always showed the highest antioxidant activities; meanwhile, *White*, *Fino de Jete* and *Campas* displayed the lowest ones, both in terms of radical scavenging and reducing activity. 

Additionally, the obtained values were positively correlated with TPC (ρ_TCP/DPPH_ = 0.983; ρ_TCP/ABTS_ = 0.940; ρ_TCP/FRAP_ = 0.928) (Figure 4), indicating that polyphenols contribute almost exclusively to the redox-active properties. Moreover, tPACs was also strongly correlated with FRAP (ρ_TPAC/FRAP_ = 0.844), but it found a lower correlation with ABTS and DPPH (ρ_TPAC/ABTS_ = 0.654; ρ_TPAC/DPPH_ = 0.664). The higher correlation found between FRAP and tPAC may be explained by the peculiar structural characteristics of proanthocyanidins, making it easier to bind metal ions thanks the presence of free meta-oriented hydroxyl groups [3,7].

Although in solution assays are widely employed to preliminarily evaluate the antioxidant capacities, they cannot measure the antioxidant activity in a biological environment [40,80]. For this purpose, cellular-based lipid peroxidation models evaluate the potential ability of redox-active compounds to interact with biological membranes [39,81,82]. Therefore, they represent interesting alternatives to in vivo models, which instead may be expensive, unethical, and not easy to use [39]. In particular, the CAA assay is a very biologically relevant method because it also takes into account the uptake, metabolism, and location of antioxidant compounds within cells [39,81]. The antioxidant activity of the ethanolic extracts of the fruits from the seven observed CVs of *A. cherimola* expressed as CAA_50_ is displayed in (Figure 6, Panel B). The average CAA_50_ value was 7.94 mg ± 1.81 of the PW per mL of cell medium was recorded. The obtained values are in the same range as those determined by Wolfe et al. under the same experimental conditions for hydrophilic extracts of other fresh fruits [81]. Among the observed CVs, a little variability in term of CAA_50_ was recorded. In particular, *Chaffey* displayed the highest activity, followed by *Daniela* and *Torre1*. Although in-solution assays are not always predictive of the antioxidant capacity in biological models, in our experimental conditions, the antioxidant activity of CAA is positively correlated with the redox-active properties evaluated by in solution assays (ρ_CAA/ABTS_ = −0.943; ρ_CAA/DPPH_ = −0.879; ρ_CAA/FRAP_ = −0.818) (Figure 4). Furthermore, the strong correlation between the CAA_50_ values and TPC (ρ_CAA/TPC_ = −0.923) and the lower correlation with tPACs (ρ_CAA/TPAC_ = −0.608) of the tested extracts suggested that the main contribution to CAA was not mainly given by the PACs, but by other polyphenol compounds. This result is not surprising, considering the low capacity of polyphenolic polymers to cross the cellular membrane [62].

### 3.5. Cultivar Discrimination via Principal Component Analysis and HeatMap Cluster Analysis

**Figure 7:**
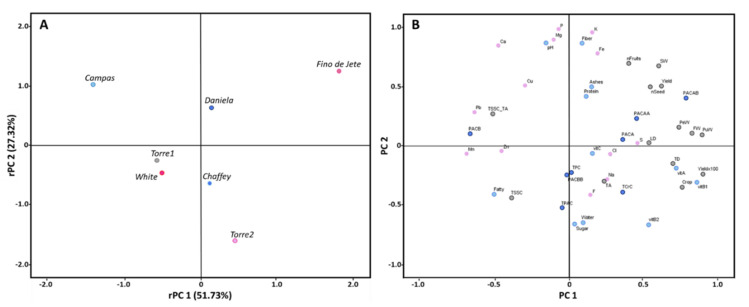
Scatter plot of the principal components factor scores of the seven observed CVs of *Annona cherimola* fruits. Panel (**A**) shows the clear separation among the different CVs; meanwhile, Panel (**B**) reports the chemical portioning of the compounds.

The Principal Component Analysis (PCA), calculated on the data matrices related to pomological, physiochemical, nutritional and nutraceutical values previously measured, allowed for the discrimination between the different fruits of the seven observed CVs of *A. cherimola* (Figure 7). In particular, PCA explained 27.32% and 51.73% of the total variance, respectively for PC1 and PC2. Positive factor scores discriminated *Fino de Jete* and *Daniela* from others CVs. In particular, *Fino de Jete* is the best CV for the highest values related to the most important commercial parameters, such as FW, LD, TD, F, and PW, but it also showed the best nutritional profile among the different CVs, having a good vitamin and mineral content. On the other hand, *Daniela* showed the best phytochemical profile and the highest antioxidant properties while having intermediate pomological, physicochemical, and nutritional values. *Campas* was completely separated from other CVs for positive PC2 and negative PC1. In particular, the separation is mainly due to the high mineral content, and to poor nutraceutical properties and very poor pomological traits. Finally, *Torre1*, *Torre2*, *White* and *Chaffey* were grouped for negative PC2. Especially, *Torre1* and *White* had also negative PC1 due to their similar pomological characteristics. On the other hand, positive PC1 and negative PC2 factor scores grouped *Torre2* and *Chaffey* for their particular PAC composition. However, *Chaffey* also had nutritional and nutraceutical properties better than *Torre2*.

**Figure 8:**
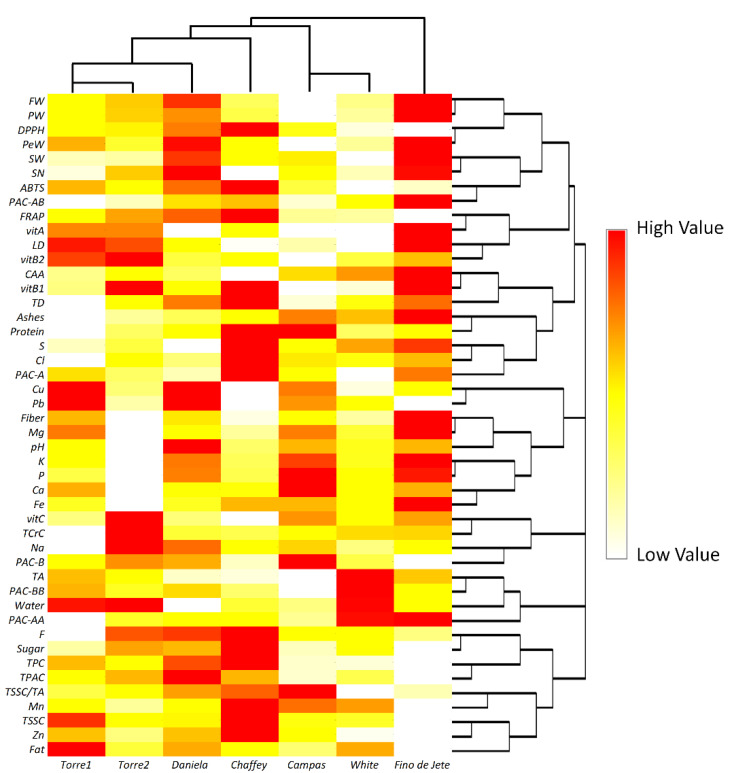
Hierarchical clustering analysis and heatmap visualization of the physiochemical, nutritional, and nutraceutical data of the seven observed cultivars of *Annona cherimola* fruits. For each row, diverse colors indicate differences between the values measured for each parameter among the seven cultivars.

The HeatMap coupled with Hierarchical Clustering Analysis confirmed the separation performed by PCA (Figure 8). In particular, *Fino de Jete* was completely separated from other CVs due to high values recorded both for some of the pomological traits, such as FW, LD, TD, PW, PeW and SN, and for the highest vitamin content. On the other hand, *White* were really far from *Fino de Jete* because the lowest values recorded for all the pomological traits of fruits, and also for the low vitamin and phytochemical content. For the same reason, *Campas* was very close to *White*. Clustering analysis revealed that the two local ecotypes, *Torre1* and *Torre2*, had not only similar pomological parameters, but they also displayed a comparable antioxidant activity both in solution and in cellular models. This proximity may also be explained by their similar PAC profile. Finally, *Daniela* and *Chaffey* take place in an intermediate position within the clustering due to their acceptable values recorded both for pomological traits, nutritional values, and nutraceutical properties.

## 4. Conclusions

In the present work, we demonstrated the overall high quality of cherimoya fruits harvested from plants grown in Sicily, in terms of pomological, physiochemical, nutritional, and nutraceutical attributes. Our results showed great variability among the seven observed CVs and may contribute to better define the potential commercial impact of the different CVs. In particular, our analysis showed *Fino de Jete* as being a commercially appreciated CV for its pomological and physiochemical attributes; it also had high nutritional values. On the other hand, the local CV *Daniela*, together to good commercial attributes, also displayed good nutraceutical properties. Concerning *White*, a CV less requested on the market, in addition to having low pomological attributes, it had reduced nutritional and nutraceutical values. Finally, the two local ecotypes, *Torre1* and *Torre2*, had qualitative attributes comparable to those measured for the international CVs.

## Figures and Tables

**Table 1:** The name of cultivar (CV), origin, yield, number of fruits per tree, yield efficiency percentage, and crop load of the observed seven *Annona cherimola* cultivars

CV	Origin	Harvest Date	Tree Vigour	Yield	Fruits Per Tree	%Yield Efficiency	%Crop Load
				(kg/tree)		(kg/cm^2^)	(fruit/cm^2^)
*Campas*	Spain	08 Nov	High	7.25 ± 0.63 ^e^	50.5 ± 3.0 ^cb^	1.01 ± 0.05 ^e^	8.12 ± 0.51 ^e^
*Chaffey*	USA-California	03 Dec	High	14.04 ± 0.64 ^c^	65.0 ± 3.0 ^b^	4.23 ± 0.19 ^c^	19.55 ± 0.47 ^a^
*Daniela*	Italy	02 Dec	Mdium-low	30.11 ± 0.12 ^a^	80.5 ± 2.0 ^a^	4.21 ± 0.21 ^c^	12.14 ± 0.43 ^c^
*Fino de Jete*	Spain	02 Dec	Medium	30.41 ± 0.78 ^a^	80.0 ± 3.0 ^a^	6.17 ± 0.26 ^a^	16.77 ± 0.21 ^b^
*Torre1*	Italy	07 Dec	Medium	17.15 ± 0.29 ^b^	45.0 ± 3.0 ^cd^	4.55 ± 0.19 ^c^	11.23 ± 0.41 ^cd^
*Torre2*	Italy	08 Nov	High	8.84 ± 0.54 ^d^	26.0 ± 2.0 ^e^	5.23 ± 0.37 ^b^	15.41 ± 0.42 ^c^
*White*	USA-California	03 Dec	High	6.83 ± 0.45 ^f^	35.5 ± 2.0 ^d^	2.41 ± 3.21 ^d^	10.11 ± 0.41 ^d^

Values are expressed as mean ± SD of data collected in two years. Among the same series, different lowercase letters indicate significantly different values at *p* ≤ 0.05, as measured by Tukey’s tests.

**Table 2:** A list of the evaluated sensory descriptors and their definitions.

Descriptors	Acronyms	Definition
**Appearance**		
Skin Color	SC	Predominant color of the main surface of the Cherimoya
Flesh Color	FC	Color of the Cherimoya flesh (from pale green to dark green)
**Aroma**		
Off-Odor	OFO	Non characteristic odor
Exotic Fruit Odor	EXO	Characteristic aroma of exotic fruit perceived with the sense of smell
Melon, Banana and Pear Odor	MBO	*Annona* characteristic odor
Medicine Odor	MO	Non characteristic odor
Grassy Odor	GO	Characteristic aroma of cut grass perceived with the sense of smell
Fruity Odor	FO	Fruit characteristic aroma
**Flavor**		
Melon, Banana and Pear Flavor	MBF	*Annona* characteristic odor
Fermented Flavor	FFL	Characteristic flavor of fruit at initial fermentation process
Exotic Fruit Flavor	EXF	Exotic fruit characteristic flavor
Fruity Flavor	FFL	Fruit characteristic flavor
Flavor Alcohol	ALF	Flavor associated with alcohol scent
Off-flavor	OFF	Non characteristic odor
**Taste and tactile in mouth**		
Acid	A	Basic taste on tongue stimulated by acids
Astringent	AST	Sensory perception in the oral cavity that may include drying sensation, and roughing of the oral tissue
Sweetness	S	Taste on the tongue stimulated by sugars and high potency sweeteners
Pungent	P	Sensation of tingling perceived in the oral cavity
**Rheological**		
Juiciness	J	The amount of juice/moisture perceived in the mouth.
Consistency	C	The force it takes to bite through the sample
General Appearance	APP	Regularity of shape, size, gloss, color and absence of defects
Mellowness	M	Perceived time during swallowing
Overall Evaluation	OVE	Overall judgment
Mealiness	MEA	A flour-like texture

**Table 3:** The pomological traits of the seven observed *Annona cherimola* fruits. Data are expressed as mean ± SD. For each row, different lowercase letters mark significant (*p* < 0.05) differences among the samples, as measured by one-way ANOVA followed by Tuckey’s test. The letter “a” denotes the highest content.

	*Campas*	*Chaffey*	*Daniela*	*Fino de Jete*	*Torre1*	*Torre2*	*White*
FW	280 ± 26.0 ^c^	392 ± 182 ^bc^	578 ± 163 ^a^	607 ± 121 ^a^	454 ± 62.6 ^b^	485 ± 71.1 ^ab^	362 ± 110 ^bc^
PW	158 ± 80.4 ^c^	290 ± 90.6 ^b^	423 ± 83.5 ^ab^	527 ± 85.5 ^a^	341 ± 90.3 ^b^	375 ± 90.8 ^b^	229 ± 45.7 ^bc^
PeW	49.8 ± 8.21 ^c^	74.7 ± 24.3 ^ab^	92.7 ± 19.9 ^a^	93.3 ± 6.70 ^a^	80.4 ± 6.80 ^ab^	70.4 ± 8.10 ^b^	59.9 ± 12.3 ^bc^
SW	16.2 ± 4.42 ^ab^	15.6 ± 10.0 ^ab^	23.5 ± 6.12 ^ab^	25.6 ± 5.81 ^a^	14.0 ± 7.00 ^ab^	14.2 ± 6.00 ^ab^	13.4 ± 3.71 ^b^
SN	31.6 ± 9.40 ^ab^	22.8 ± 14.3 ^b^	45.0 ± 6.81 ^a^	44.5 ± 17.7 ^a^	24.0 ± 11.1 ^ab^	34.3 ± 7.80 ^ab^	25.3 ± 5.12 ^ab^
LD	85.9 ± 11.3 ^c^	78.3 ± 9.70 ^c^	102 ± 11.9 ^b^	120 ± 15.5 ^a^	119 ± 10.7 ^ab^	115 ± 16.0 ^ab^	77.5 ± 9.30 ^c^
TD	80.3 ± 4.20 ^b^	103 ± 9.61 ^a^	98.4 ± 6.90 ^a^	98.9 ± 6.10 ^a^	77.8 ± 5.00 ^b^	93.5 ± 7.61 ^a^	92.8 ± 12.1 ^a^
FF	1.01 ± 0.60 ^ab^	1.90 ± 1.51 ^a^	1.72 ± 0.40 ^ab^	0.81 ± 0.90 ^b^	0.60 ± 0.51 ^b^	1.60 ± 0.41 ^ab^	1.01 ± 1.11 ^ab^

FW is the Fruit Weight; PW is the Pulp Weight; PeW is the Peel Weight; SW is the Seed Weight; SN is the Seed Number; LD is the Longitudinal diameter; TD is the Transversal diameter; F is the firmness.

**Table 4:** Color and physiochemical parameters of the seven observed *Annona cherimola* fruits. For each parameter, different letters indicate significant (*p* < 0.05) differences among the cultivars as measured by one-way ANOVA followed by Tuckey’s test. Letter “a” denotes the highest content.

**Colour Parameters**								
	**Peel Colour of Fruits**				**Pulp Colour of Fruits**			
	L*	a*		b*	L*	a*		b*
*Campas*	61.1 ± 4.61 ^bc^	−11.9 ± 1.71 ^c^		30.0 ± 2.82 ^a^	78.4 ± 3.81 ^a^	−1.06 ± 0.85 ^ab^		15.9 ± 2.02 ^a^
*Chaffey*	57.6 ± 4.82 ^c^	−8.88 ± 1.82 ^ab^		29.5 ± 2.92 ^a^	82.8 ± 2.91 ^a^	−0.92 ± 0.31 ^ab^		13.7 ± 1.83 ^ab^
*Daniela*	59.1 ± 3.71 ^bc^	−11.0 ± 2.21 ^b^		29.5 ± 2.33 ^a^	81.4 ± 5.69 ^a^	−1.06 ± 0.54 ^ab^		14.6 ± 2.02 ^ab^
*Fino de Jete*	69.6 ± 2.72 ^a^	−8.73 ± 1.62 ^a^		30.6 ± 2.64 ^a^	83.5 ± 2.78 ^a^	−1.14 ± 0.22 ^ab^		13.0 ± 1.74 ^b^
*Torre1*	63.2 ± 4.01 ^b^	−11.5 ± 1.93 ^bc^		31.1 ± 3.19 ^a^	82.0 ± 2.69 ^a^	−1.53 ± 0.25 ^b^		12.0 ± 1.88 ^b^
*Torre2*	63.1 ± 1.48 ^b^	−10.3 ± 2.55 ^b^		30.2 ± 1.97 ^a^	82.3 ± 3.39 ^a^	−0.91 ± 0.24 ^ab^		13.9 ± 1.59 ^ab^
*White*	59.7 ± 2.90 ^bc^	−10.5 ± 1.41 ^b^		30.4 ± 1.02 ^a^	81.3 ± 3.91 ^a^	−0.81 ± 0.24 ^a^		13.3 ± 1.58 ^b^
**Physiochemical Parameters**								
	**TSSC**		**TA**		**TSSC/TA**		**pH**	
	**(°Brix)**		**(g L^−1^ of malic acid)**					
*Campas*	18.9 ± 1.01 ^b^		3.57 ± 0.81 ^c^		7.56 ± 7.40 ^a^		5.23 ± 0.91 ^bc^	
*Chaffey*	22.2 ± 4.02 ^a^		3.69 ± 0.70 ^c^		6.67 ± 2.61 ^a^		4.64 ± 0.33 ^c^	
*Daniela*	19.1 ± 1.32 ^b^		3.76 ± 0.40 ^c^		6.08 ± 3.40 ^a^		5.75 ± 0.44 ^ab^	
*Fino de Jete*	16.9 ± 0.81 ^b^		4.61 ± 0.80 ^ab^		4.48 ± 2.50 ^b^		5.23 ± 0.45 ^bc^	
*Torre1*	21.6 ± 1.33 ^a^		4.64 ± 0.32 ^ab^		4.91 ± 1.50 ^ab^		5.01 ± 0.31 ^bc^	
*Torre2*	19.0 ± 0.84 ^b^		4.43 ± 0.71 ^ab^		5.18 ± 2.81 ^ab^		6.02 ± 1.42 ^a^	
*White*	18.7 ± 1.01^b^		5.26 ± 0.40 ^a^		4.18 ± 2.51 ^b^		4.90 ± 0.11 ^c^	

TSSC is the Total Solid Soluble Content; TA is the Titratable Acidity; TSSC/TA is the ratio between the Total Solid Soluble Content; L* is the lightness; a* is the variation from red (+a*) to green (−a*); b* is the variation from green (+b*) to yellow (−b*). Data are mean values ± SD.

**Table 5:** The nutritional, mineral, and vitamin composition of the seven observed *Annona cherimola* fruits. Data are mean values ± SD, and they are expressed per 100 of Pulp Weight (PW). For each row, different letters mark statistical (*p* < 0.05) differences among the cultivars, as measured by one-way ANOVA analysis followed by Tuckey’s test. The letter “a” denotes the highest content.

	*Campas*	*Chaffey*	*Daniela*	*Fino de Jete*	*Torre1*	*Torre2*	*White*
**Nutritional Content** *(g per 100 g of PW)*							
Water Content	78.4 ± 0.39 ^ab^	78.9 ± 0.68 ^ab^	77.5 ± 0.61 ^b^	79.3 ± 0.93 ^ab^	80.3 ± 0.63 ^a^	80.4 ± 1.26 ^a^	80.3 ± 0.91 ^a^
Ashes	0.70 ± 0.07 ^a^	0.68 ± 0.07 ^ab^	0.63 ± 0.07 ^ab^	0.72 ± 0.02 ^a^	0.53 ± 0.01 ^b^	0.59 ± 0.01 ^ab^	0.69 ± 0.06 ^ab^
Protein Content	1.75 ± 0.10 ^a^	1.75 ± 0.13 ^a^	1.69 ± 0.08 ^ab^	1.69 ± 0.18 ^ab^	1.31 ± 0.11 ^b^	1.56 ± 0.13 ^ab^	1.56 ± 0.12 ^ab^
Fat Content	0.19 ± 0.04 ^ab^	0.23 ± 0.01 ^a^	0.25 ± 0.01 ^a^	0.14 ± 0.01 ^b^	0.29 ± 0.02 ^ab^	0.21 ± 0.01 ^a^	0.25 ± 0.02 ^a^
Sugar Content	13.0 ± 1.31 ^ab^	16.0 ± 0.57 ^a^	14.7 ± 2.09 ^ab^	12.5 ± 0.89 ^b^	13.1 ± 0.53 ^ab^	14.9 ± 1.35 ^ab^	14.2 ± 0.49 ^ab^
Raw Fibber Content	4.32 ± 0.11 ^a^	2.35 ± 0.08 ^b^	4.41 ± 0.49 ^a^	5.35 ± 0.26 ^a^	4.61 ± 0.95 ^a^	2.11 ± 0.07 ^b^	2.93 ± 0.07 ^b^
Energy *(KJoule)*	254 ± 8.94 ^c^	306 ± 5.62 ^a^	284 ± 3.66 ^ab^	243 ± 3.53 ^d^	254 ± 8.63 ^c^	283 ± 8.01 ^ab^	274 ± 9.01 ^b^
**Mineral Content** *(mg per 100 g of PW)*							
K	273.3 ± 13.2 ^ab^	169 ± 6.50 ^c^	246 ± 25.0 ^b^	303 ± 34.6 ^a^	182 ± 4.35 ^c^	146 ± 11.3 ^c^	178 ± 3.63 ^c^
Na	20.0 ± 7.02 ^a^	19.3 ± 6.42 ^a^	21.6 ± 9.60 ^a^	19.3 ± 5.03 ^a^	17.3 ± 1.15 ^a^	23.3 ± 10.0 ^a^	18.3 ± 4.72 ^a^
Ca	10.0 ± 1.01 ^a^	9.00 ± 1.02 ^a^	9.01 ± 1.73 ^a^	9.33 ± 1.15 ^a^	9.33 ± 1.15 ^a^	8.01 ± 2.12 ^a^	9.02 ± 1.01 ^a^
Mg	10.0 ± 1.11 ^a^	8.66 ± 0.57 ^a^	9.66 ± 2.51 ^a^	10.3 ± 0.57 ^a^	10.1 ± 2.02 ^a^	8.02 ± 1.03 ^a^	9.33 ± 1.15 ^a^
P	31.6 ± 2.08 ^a^	22.5 ± 9.19 ^a^	28.0 ± 3.02 ^a^	31.0 ± 3.03 ^a^	22.6 ± 2.08 ^a^	18.2 ± 5.29 ^a^	24.3 ± 2.51 ^a^
S	20.6 ± 2.08 ^a^	25.3 ± 4.93 ^a^	19.3 ± 4.72 ^a^	24.3± 2.08 ^a^	19.6 ± 3.51 ^a^	20.3 ± 4.52 ^a^	22.3 ± 2.08 ^a^
Cl	88.0 ± 12.5 ^a^	96.0 ± 9.64 ^a^	80.0 ± 6.55 ^a^	89.6 ± 17.4 ^a^	71.6 ± 1.52 ^a^	87.3 ± 9.29 ^a^	86.6 ± 1.52 ^a^
Cu	0.16 ± 0.09 ^a^	0.09 ± 0.02 ^a^	0.18 ± 0.06 ^a^	0.13 ± 0.02 ^a^	0.19 ± 0.06 ^a^	0.13 ± 0.07 ^a^	0.09 ± 0.05 ^a^
Mn	0.11 ± 0.02 ^a^	0.12 ± 0.01 ^a^	0.10 ± 0.02 ^a^	0.08 ± 0.06 ^a^	0.10 ± 0.03 ^a^	0.09 ± 0.07 ^a^	0.11 ± 0.01 ^a^
Zn	0.51 ± 0.05 ^a^	0.56 ± 0.08 ^a^	0.52 ± 0.06 ^a^	0.38 ± 0.02 ^a^	0.52 ± 0.06 ^a^	0.44 ± 0.09 ^a^	0.39 ± 0.01 ^a^
Fe	0.34 ± 0.01 ^ab^	0.34 ± 0.06 ^a^	0.31 ± 0.01 ^ab^	0.39 ± 0.03 ^a^	0.31 ± 0.01 ^ab^	0.24 ± 0.03 ^b^	0.32 ± 0.04 ^ab^
**Vitamin Content** *(mg per 100 g of PW)*							
Retinol (A)	n.d.	0.14 ± 0.01 ^a^	n.d.	0.31 ± 0.02 ^ab^	0.22 ± 0.02 ^ab^	0.22 ± 0.01 ^b^	n.d.
Thimine (B1)	0.05 ± 0.01 ^ab^	0.13 ± 0.01 ^a^	0.11 ± 0.01 ^ab^	0.13 ± 0.01 ^ab^	0.08 ± 0.01 ^ab^	0.13 ± 0.02 ^b^	0.06 ± 0.01 ^b^
Rboflvin (B2)	0.08 ± 0.01 ^a^	0.12 ± 0.01 ^a^	0.11 ± 0.01 ^a^	0.13 ± 0.01 ^a^	0.15 ± 0.01 ^a^	0.16 ± 0.01 ^a^	0.11 ± 0.01 ^a^
Ascorbic Acid (C)	43.2 ± 0.85 ^ab^	25.4 ± 0.46 ^abc^	32.2 ± 0.35 ^abc^	42.6 ± 0.94 ^a^	31.9 ± 0.83 ^bc^	50.1 ± 1.98 ^c^	38.2 ± 0.75 ^c^
